# Implementation of an integrated community approach in deprived neighbourhoods: a theory-based process evaluation using the Consolidated Framework for Implementation Research (CFIR)

**DOI:** 10.1177/14034948231199804

**Published:** 2023-09-19

**Authors:** Sanneke J.M. Grootjans, M.M.N. Stijnen, I. Hesdahl-De Jong, M.E.A.L. Kroese, D. Ruwaard, M.W.J. Jansen

**Affiliations:** 1 Department of Health Services Research, Care and Public Health Research Institute (CAPHRI), Faculty of Health, Medicine and Life Sciences, Maastricht University, Maastricht, the Netherlands; 2 Academic Collaborative Centre for Public Health Limburg, Public Health Service South Limburg (GGD Zuid Limburg), Heerlen, the Netherlands

**Keywords:** Consolidated Framework for Implementation Research, deprived neighbourhoods, integrated care, integrated community approach, multi-professional collaboration, population health management

## Abstract

**Background:**

We investigated the implementation process of an Integrated Community Approach (ICA) applied in four low socio-economic status neighbourhoods in Maastricht, the Netherlands. The ICA is a Population Health Management initiative and aims to improve population health, quality of care, professional’s satisfaction and decrease costs of care. This study addresses the facilitators and barriers for implementing the ICA from a stakeholder perspective, including steering group members, professionals and citizens.

**Methods:**

We conducted a mixed-methods study using a triangulation of methods to investigate the implementation from 1 December 2016 to 31 December 2020. The Consolidated Framework for Implementation Research guided data collection and data-analysis for evaluating the implementation process. In total, 77 interviews, 97 observations, seven focus groups, 65 collected documents and two surveys with open-ended questions were conducted.

**Results:**

Facilitators for implementation were the use of citizen science to bring residents’ needs into sharp focus, the integration of the ideology of Positive Health into the working routines of the professionals and leadership at the steering group level to overcome barriers in the ICA. The existing accounting and financial infrastructure obstructed combining budgets at neighbourhood level.

**Conclusions:**

**Engaging citizens and professionals at an early stage is an important facilitator for implementation. The use of a shared vision on health also worked as a facilitator since it created a shared language among professionals, which is important in Population Health Management initiatives where multiple professionals are expected to collaborate**.

**Trial Registration:**

NTR 6543; registration date, 25 July 2017.

## Introduction

Many Western countries are confronted with the consequences of population ageing and complex health concerns. High rates of people suffering from complex and/or multiple (chronic) health complaints and related health care expenditures call for the improvement of health outcomes, which cannot be achieved by the healthcare sector alone [1]. Population Health Management (PHM) strategies are advocated as a possible solution. PHM focusses on integrating and reorganising public health, healthcare, social care and community services with the ultimate aim of improving population health and enhancing quality of care while decreasing the cost of care in a defined group of individuals [2], also known as the ‘Triple Aim’ [3]. Bodenheimer and Sinsky [4] expanded the goals to include improving provider satisfaction, resulting in the ‘Quadruple Aim’. The transition to a health and wellbeing system initiated by PHM strategies is in line with one of the Sustainable Development Goals of the United Nations (UN) 2030 Agenda for Sustainable Development, namely, to ensure healthy lives and promote wellbeing for all at all ages [5].

In many European countries, including the Netherlands, PHM initiatives have been developed and implemented in different settings and in collaboration with a broad range of cross-sector stakeholders [6]. The Dutch Ministry of Health, Welfare and Sport aimed to drive collaboration and to develop a sustainable healthcare system in the Netherlands. Therefore, nine Dutch regions were selected in 2013 as pioneer sites to experiment with PHM [7]. One of these sites is ‘Blue Care’ (representing ‘sustainable care’, equivalent to using ‘green’ for sustainable environments) in the southern part of the Dutch province of Limburg. ‘Blue Care’ was initiated by the primary care organisation (‘ZIO’), health care insurer (‘VGZ’), Maastricht University Medical Centre (MUMC+) and a patient organisation (‘Burgerkracht Limburg’). Collaboration in the ‘Blue Care’ pioneer site was extended to health care organisations, care and well-being providers in the social domain, social services, public health services and Maastricht University. All partners committed themselves to achieving the Quadruple Aim goals.

The present study focusses on one of the initiatives developed as part of the ‘Blue Care’ pioneer site: the Blue Care integrated community approach (ICA), implemented in four low socio-economic status (SES) neighbourhoods in the municipality of Maastricht [8]. This study investigated the implementation of the ICA from the perspective of the stakeholders (i.e. financial sponsors, daily board members, ambassadors and project team members) and health and social care professionals involved in the ICA and the citizens living in the neighbourhoods.

These kinds of population health initiatives are perceived as complex since they involve complex multifactorial aetiology and are strongly influenced by context [9,10]. A process evaluation may offer insight in the underlying mechanism of implementation and may help to identify factors which are responsible for failure of success of an intervention, including the context [11]. Using a theoretical framework to guide data collection, analysis and interpretation is crucial to identify determinants of implementation that are relevant also outside the specific context in which the research was conducted [12]. Therefore, in this study, we used the Consolidated Framework for Implementation Research (CFIR) to guide data collection and analysis [13].

The research question addressed is: what are the facilitators and barriers for implementing the Blue Care ICA between 2016 and 2020 in four low socio-economic status neighbourhoods in the city of Maastricht, the Netherlands?

## Methods

### Study design

We conducted a mixed-methods process evaluation using a triangulation of methods to investigate the implementation of an ICA that was developed and implemented from 1 December 2016 to 31 December 2020 [14]. Using a convergent-parallel approach, quantitative and qualitative methods were of equal importance and complemented each other to understand the context and complexity of the implementation process [15]. Data collection and coding of the data were conducted with qualitative research methods. After coding the data, valence and strength in the found elements among the CFIR constructs were rated in a quantitative manner to generalize the data for future research.

### Integrated community approach

The ICA is based on the ideology of ‘Positive Health’, where health is described as ‘the ability to adapt and self-manage in the face of social, physical, and emotional challenges’ [16]. This definition of health goes beyond focusing on disease and illness by also including the individual’s perceived sense of control and ability to cope with life events. Next to using it as a shared ideology, Positive Health offers a practical dialogue tool (called ‘the spiderweb’ instrument) intended to guide person-centred interactions and decision-making between health professionals and clients or patients. Health care and social care professionals involved in the ICA were trained in Positive Health and the application of the dialogue tool with clients in practice. Besides expected benefits for patients or clients, adopting Positive Health may contribute to the job satisfaction of professionals as evidenced by a preliminary study among primary care professionals in general practice.

Core elements of the ICA are as follows.

1. Citizens in action: apply a bottom-up approach, instead of top-down, in which citizens’ perspectives and needs are the starting point for change and/or for projects to tackle bottlenecks as experienced by citizens.
2. Professionals in action: enable and support the professional freedom of health and social care professionals working in the four neighbourhoods to organise (preventive) support in the main interest of the individual citizen, above and beyond organisational interests or financial reasons.
3. Combining budgets: build a reimbursement system in which the budgets for health and social care are combined for the four neighbourhoods at the population level. The Dutch health care system is governed by four basic health-care-related acts: the Health Insurance Act, the Long-Term Care Act, the Social Support Act and the Youth Act. The Health Insurance Act (curative medicine, e.g. hospital care) and the Long-Term Care Act (permanent or 24-h home care) account for the majority of the healthcare budget available in the Netherlands. Local authorities (municipalities) are responsible for implementing the Social Support Act and the Youth Act, which involve support, assistance or care services by a health care provider (for more details, see Ministry of Health, Welfare and Sport) [17]. The ICA aims to combine the budgets of the municipality of Maastricht with the budgets of the dominant health insurer for citizens living in Maastricht.

**Figure 1.**
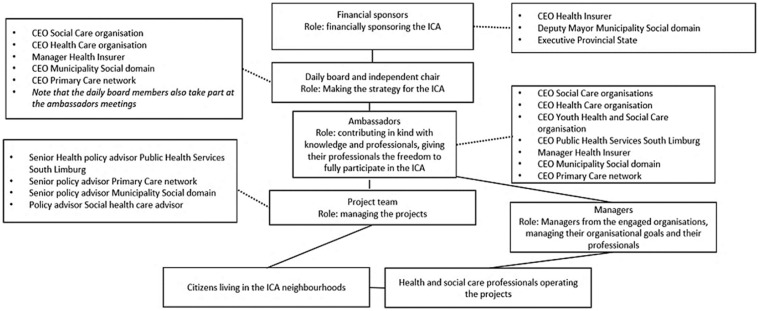
Stakeholders involved in the ICA. CEO: chief executive officer; ICA: integrated community approach.

An overview of the stakeholders involved in the ICA, including the governance structure, is shown in Figure 1. Cross-sector collaborations and partnerships are considered as essential components in PHM, but also require a considerable investment in time and effort to create shared commitment, mutual understanding and trust, especially in the starting phase [18]. The characteristics of collaborative governance that facilitated or hampered collaboration in the ICA in the starting phase are described elsewhere [19].

### Projects

In the ICA, several projects were developed ‘on the go’. The projects originated from the bottlenecks in the delivery of care and welfare support as experienced by professionals (e.g. general practitioners (GPs), home care nurses, social workers) and citizens. Eight main projects were developed, ranging from Positive Health training to coaching families with high health and social care usage.

### Setting

The ICA was implemented in four low SES neighbourhoods (Limmel, Nazareth, Wittevrouwenveld, Wyckerpoort) located in Maastricht, the Netherlands. Citizens living in these four neighbourhoods (*n* = 15,290 inhabitants) are less healthy and socioeconomically deprived compared with the rest of Maastricht (*n* = 122,144 inhabitants) [20]. The four neighbourhoods are the working area of two GP practices and of health and social care providers from more than 50 organisations. In the Dutch health care system, the GP is the gatekeeper to specialised medical services financed by the health insurer. Access to social care and support is organised at the municipal level, meaning that municipalities are responsible for social services such as psychosocial support and home care. They can offer these services themselves or outsource them to private welfare organisations [17]. Social district teams consisting of various professionals (e.g. home care nurses, Social Support Act consultants and social workers) organise access to social care at neighbourhood level for citizens. One team is active in the neighbourhoods of Limmel and Nazareth and one team in the other two neighbourhoods.

### Data collection

We used the CFIR to guide the data collection and data analysis for evaluating the implementation process of the ICA from 1 December 2016 to 31 December 2020. The CFIR is a conceptual framework that offers a pragmatic structure for identifying potential influences on implementation and can be used to build knowledge about what works where, across multiple settings. The CFIR can be considered as a ‘determinants framework’, which can identify barriers and facilitators using deductive reasoning [21]. It comprises five major domains: intervention characteristics, outer setting, inner setting, characteristics of the individuals involved and the process of implementation. These are in turn divided into 39 subdomains attempting to cover the complex context of the implementation process. The CFIR model can be used for multiple evaluation types [13]. The data collection methods are described in detail below. In total, 77 interviews, 97 observations, seven focus groups, 65 collected documents and two surveys with open-ended questions were conducted.

### Interviews

Interviews were conducted in 2017 (*n* = 2 financial sponsors, *n* = 5 daily board), 2018 (*n* = 17 citizens, *n* = 3 financial sponsors, *n* = 5 daily board, *n* = 1 independent chair, *n* = 4 ambassadors, *n* = 4 managers, *n* = 4 project team, *n* = 4 professionals), 2019 (*n* = 9 managers, *n* = 12 professionals and *n* = 3 project team) and 2020 (*n* = 4 daily board). The number of stakeholders interviewed per year varied, as the interviews were used to address specific aspects of implementing the ICA that arose during the field observations and document reviews. All interviews were audio recorded and transcribed verbatim. The mean duration of the interviews was 45 min. Informed consent was obtained from all stakeholders. The domains of the CFIR model formed the interview guide. The same interview guide was used from 2017 to 2020, while also allowing the interviewees to express additional opinions and viewpoints.

### Observations

Observations were conducted during existing meetings between stakeholders in the ICA between 1 December 2016 and 31 December 2020. The meetings observed were six financial sponsor meetings, 24 daily board meetings, seven ambassador meetings, 39 project team meetings, 12 meetings with the professionals in the field and nine observations in the neighbourhoods’ community centres and on the street. We used the domains of the CFIR model as an overarching guide for the observations since the themes of the meetings varied between the stakeholders. The observations provided insight into how the process of implementation of the ICA evolved over time. Notes of these observations were documented in a word file with the date, who was observed and what was observed.

### Focus groups

Seven focus groups were conducted, of which four were organised with professionals involved in the ICA and three with citizens. The focus groups were a valuable addition to the interviews, as they provided insight into group dynamics and interprofessional collaboration. The domains of the CFIR were used as an overarching topic guide for the focus groups with the professionals. For the focus groups with the citizens, we used the items of the practical dialogue tool of Positive Health as a topic guide. The rationale for the focus groups was to understand how the ICA was implemented on the professional and citizen level and what their role was [22]. Two focus groups with professionals took place in 2018 (*n* = 11 and *n* = 12 participants) and two in 2020 (*n* = 10 and *n* = 11 participants). The focus groups in 2020 took place online because of COVID-19 measures. Participating professionals were the members of the social districts’ teams in the four neighbourhoods. The three focus groups with citizens both took place in 2019 (*n* = 2, *n* = 3 and *n* = 7 participants) with the citizens from the citizen science trajectory as participants. The focus groups were audio recorded and transcribed verbatim.

### Documents

We collected 65 documents. The documents consisted of 30 official minute notes, 17 reports written by stakeholders involved in the ICA, two magazines with information about the projects of the ICA and (preliminary) results per project and 16 other media files, such as a newspaper article and media interviews with stakeholders.

### Survey

Open questions were used of a post-measurement online survey distributed in 2020 among professionals (e.g. GPs, social health workers, home care nurses), policymakers and managers trained in the concept of Positive Health. The response rate was 32.6% (*n* = 62) at the first measurement and 26.1% (*n* = 46) at the second measurement. The questions involved how the professionals use Positive Health in daily practice and open remarks about their experience with Positive Health.

### Data analysis

All transcripts and documents were included in the NVivo 12 software program. We analysed all data together without distinguishing between professions or source method since the focus of this paper is on process and implementation of the ICA as a whole. We had no hierarchical strategy for the different data sources, but used them to enhance each other to create a nuanced understanding of the data. The coding was carried out by two researchers (SG and IH). Content analysis was used to identify patterns and themes in our data [23], which we coded in the existing codebook of the CFIR [12]. We started with open coding the data by reading through the data and identifying relevant segments of text (initial codes). Each researcher then reviewed the initial codes and grouped them into themes, which were then refined into a smaller set of key themes. Key themes and underlying codes were compared between the researchers. In case of major differences, a third researcher (MS) was consulted. After consensus was reached, SG and IH linked the key themes to the CFIR constructs. We constantly revised the coding scheme to ensure that it captured the meaning of our data.

To increase intercoder reliability, the researchers discussed the codebook beforehand to specify and clarify the codes and to make sure the codes were interpreted equally by both researchers. To increase the trustworthiness of the coding, the researchers randomly selected five documents to code blind from each other and compare them. We assessed the intercoder reliability between the researchers with the intercoder reliability calculator tool of the NVivo 12 software program, resulting in a level of agreement of 85%, indicating a substantial level of consistency in the coding process.

To stimulate comparison across cases and studies, CFIR constructs can be scored quantitatively according to strength and valence [21]. After the general coding and analysis of the data, we rated the CFIR constructs per core element of the ICA (citizens in action; professionals in action; combining budgets) using the general rating rules of the CFIR model (i.e. −2 is a negative influence on implementation (barrier); +2 is a positive influence on implementation (facilitator)).We did this by evaluating the construct in terms of facilitating or hampering factors we found in the data. The researchers both independently rated the constructs and compared them. When ratings differed between the researchers, differences were discussed with a third researcher (MS) to reach consensus.

When comments were mixed, we tipped the rating to a weak negative −1 or weak positive +1 rating. When comments were equally positive and negative, we rated the construct with an ‘X’. When the construct was not relevant or present in our data, we rated it with a ‘−’. We only rated the constructs relevant to our research question.

## Results

Table I presents an overview of the ratings assigned per CFIR construct for each of the core elements of the ICA separately. We excluded the CFIR constructs which were not relevant or present in our data for all three core elements (rating ‘−’), resulting in eight constructs that were excluded. To give more meaning and understanding of our data, we included quotes in our paper. The quotes were selected on their ability and comprehensiveness to give meaning to the construct and core elements.

**Table I.** Rating of the CFIR constructs per core element of the ICA.

Five domains with their constructs	Core element: citizens in action	Core element: professionals in action	Core element: combining budgets
**1. Innovation characteristics**			
Innovation source	−2	+2	−
Relative advantage	+1	X	+2
Adaptability	−	+2	−
Trialability	−	+1	−2
Complexity	−	−	−2
Cost	−	+1	+1
**2. Outer setting**			
Needs and resources of those served by the organisation	+2	+2	−
Cosmopolitanism	−	−2	−
**3. Inner setting**			
Networks and communications	−1	−1	−
Implementation climate	−1	+1	−2
Tension for change	+1	+2	−
Compatibility	−	−1	−
Relative priority	−	−1	−
Learning climate	−	+2	−
(readiness for implementation) Leadership engagement	−	−	+2
(readiness for implementation) Available resources	−	−	+1
(readiness for implementation) Access to knowledge and information	−	+1	−
**4. Characteristics of individuals**			
Knowledge and belief about the innovation	−	−1	−
Self-efficacy	+1	−	−
Individual stage of change	−	+1	−
Individual identification with organisation	−	+1	−
Other personal attributes	−	+1	−
**5. Process**			
Planning	−	+1	−
Engaging	+1	+2	−
Opinion leaders	+2	+2	−
Champions	−	+2	−
Key stakeholders	+1	+1	−
Innovation participants	+2	−	−
Reflecting and evaluating	−	+1	+1

−2: barrier; −1: weak barrier; X: equal ratings barrier/facilitator; +1: weak facilitator; +2: facilitator; −: not relevant or present in our data to answer the research question.

CFIR: consolidated framework for implementation research; ICA: integrated community approach.

### Innovation characteristics

#### Citizens in action

Citizens perceived the ICA as externally developed in the starting phase. The main reason for this was that the citizens were not involved in the creation of the projects in the starting phase (‘innovation source’). This changed when Positive Health was introduced and citizens came into contact with it through projects. Citizens perceived Positive Health as an advantage (‘relative advantage’) over the common vision on health:

Positive Health looks at health in a broad way, just how it’s in real life. For example, when you have financial troubles, this affects your health as well. (Citizen Scientist, interview 2018)

#### Professionals in action

Additionally, the professionals working in the neighbourhoods initially perceived the ICA as externally developed. This changed over time to internally developed once the training in Positive Health and implementation of the projects started (‘innovation source’). With the implementation of the projects, the professionals became more engaged in the design and the execution of the projects, which increased ownership of the ICA (‘adaptability’). The implementation of Positive Health was perceived as an advantage by the professionals (‘relative advantage’):

I hope with my whole heart that we keep using the ideology of Positive Health. In my opinion, we entered a road where we cannot turn around anymore. (Professional, open quote in survey 2020).

A barrier perceived by professionals was that only the professionals from participating organisations in the ICA worked according to the principles of Positive Health, while the neighbourhoods were also the working areas of other organisations outside the ICA (‘trialability’).

#### Combining budgets

In the starting phase of the ICA, combining budgets was seen as a significant advantage opposed to keeping the existing financial structure (‘relative advantage’). However, in practice this was not achievable within the existing structures (‘trialability’ and ‘complexity’). In 2016 and 2017, there was a great deal of discussion among the stakeholders about how the combined budget would operate. These discussions slowed the implementation process. In the beginning of 2018, the stakeholders jointly decided to start the implementation of the ICA projects without a combined budget. They made a verbal agreement, based on trust, to equalise odd costs between health and social care and between participating organisations (‘cost’):

We did not create a combined budget, it’s too complex, but we just had to start. . .However, I do trust that if there are major differences in our budgets, we will compensate each other. (Board member, interview 2018)

### Outer setting

#### Citizens in action

The overall health needs of the citizens living in the ICA neighbourhoods on a population level (‘needs and resources’) were not visible in the starting phase of the ICA, which hindered implementation:

We know that the health of the citizens is poorer than the rest of Maastricht, but do we know what they (the citizens) think they need to solve this? (Member Project Team, quote from observation, 2018)

Therefore, in 2018 a ‘citizen science’ trajectory started, aimed at identifying the needs of the citizens living in the four neighbourhoods by the citizens themselves.

#### Professionals in action

Knowing the bottlenecks in the delivery of care and welfare support worked as a facilitator for the professionals (‘needs and resources’), as they were the starting ground for the projects developed in the ICA (document, 2017). The ICA consisted of a network of multiple organisations and stakeholders who signed a commitment form (‘cosmopolitanism’). The four neighbourhoods were also the working areas of other, smaller organisations not involved in the ICA, which initially slightly hindered implementation. For example, in the project ‘multi-problem families’, multiple caregivers from different organisations were involved in offering health and social care to one family:

It’s difficult to get all the caregivers around the table for a multidisciplinary meeting. In one of the families I support 15 health and social care givers are active. Some of them don’t even want to meet since they don’t see the point, they’re not part of the ICA so why should they. (Professional working in the project ‘multi-problem families’, quote from observation 2019)

### Inner setting

#### Citizens in action

Plans were made in the starting phase to launch a marketing campaign in the neighbourhoods to create awareness about the ICA and Positive Health among citizens and professionals (‘networks and communications’). However, this never really got off the ground due to time constraints of the project team members and budget limitations. Professionals communicated with the citizens about the concrete content of the ICA which enhanced implementation. The four ICA neighbourhoods were, in the past, also used in the design of other health interventions. Citizens were therefore sceptical about the ICA, especially in the starting phase (‘implementation climate’). Eventually, citizens recognised the urgency of improving the deprived health status of the citizens living in the neighbourhoods, which worked as a facilitator for implementation (‘tension for change’):

Although I’m really proud of my neighbourhood, I also see that people are living unhealthy and that there’s more aggression and poverty on the streets. We need to change this. (Citizen, interview 2017)

#### Professionals in action

On a professional level, communication about the meaning of the ICA was indistinct in the starting phase (‘networks and communication’). In particular, the name of the ICA ‘Blue Care’ and overarching goal were confusing. The meaning of the ICA became clearer to professionals once the ICA communicated about the start of concrete projects in a magazine and through a newsletter which all stakeholders received (‘access to knowledge and information’). For the professionals, fragmented care and ‘pillar thinking and acting’ of health and social care professionals were seen as aspects that needed to change (‘tension for change’). In order to make the ICA principles fit with the professionals and their mother organisations (‘compatibility’), the professionals were granted freedom from their mother organisations to work according to the principles of Positive Health and to ‘think’ outside their own domain if necessary. In practice, the compatibility with existing workflows was a barrier in the starting phase. Especially at the tactical (managers) and operational levels (professionals), implementing elements or projects of the ICA received lower priority (‘relative priority’) as some organisations were already implementing other innovations or systems. The training and coaching in Positive Health for the professionals worked as a facilitating factor for a healthy learning climate (‘learning climate’). After the training in Positive Health, the professionals were coached by two coaches of the ICA to discuss any barriers in the use of Positive Health in practice:

I coach professionals with the use of Positive Health. I am a professional myself, so I know what the difficulties can be in practice. (Professional, interview 2019)

#### Combining budgets

The ‘implementation climate’ was not ready for a reimbursement system in which the budgets for health care and social care could be combined for the four neighbourhoods at a population level. The financial sponsors each contributed a certain budget per year to the ICA (source: official budget documents of the ICA). The participating organisations contributed ‘in-kind’ to the ICA in knowledge and staff hours (‘available resources’). A financial contribution to the ICA by all stakeholders facilitated implementation.

Variety in the way the three financial sponsors contributed created discussion and hindered implementation. The Provincial State and the municipality both sponsored a lump sum amount annually. The health insurer sponsored goal-oriented projects, which they believed made a good business case in reducing health care costs. The difference in sponsorship and the health insurer’s prerequisites regarding cost saving of projects created several ‘go or no-go’ moments among the financial sponsors throughout the life-span of the ICA.

One of the board members stood out for his leadership to overcome implementation and budgetary difficulties and worked as a mediator between the board and the financial board members of the ICA (‘leadership engagement’):

I’m not afraid to state that if I did not make all these (unofficial) phone calls we would not have an ICA anymore. (Board member, interview 2018)

### Characteristics of individuals

#### Citizens in action

One of the aims of the citizen science trajectory was to empower the citizens themselves. In the beginning of the project, the citizen scientists where insecure in what they could bring to the table. Nevertheless, their confidence in benefiting the health of their community and belief in their own capabilities grew (‘self-efficacy’):

I’m starting a vlog about health to reach more people. I wanted to do this but was too shy before. Now I know I can do this. (Citizen scientist, quote from observation, 2019)

#### Professionals in action

The professionals needed time to understand the values, facts and principles of the ICA (‘knowledge and belief about the innovation’). We saw that enthusiasm, skills and use of the ICA grew over time among the professionals (‘individual stage of change’). When more projects emerged, the ICA became much more tangible for the professionals. Additionally, the ICA received media attention from the rest of the Netherlands (the Dutch King visited the four neighbourhoods), and this facilitated the enthusiasm among the professionals about the ICA:

The Dutch King is visiting our project and neighbourhoods, I’m proud to be part of this. (Professional, quote from observation, 2019)

We found facilitators and barriers in the broad construct of ‘individual identification with the organisation’. There were stakeholders and organisations strongly committed to the ICA and also less committed ones. Furthermore, differences in motivation, values and competence were observed (‘other personal attributes’). There were professionals who were active in the ICA and contributed to the development of the ICA and there were more neutral ones. The ‘neutral’ professionals had no strong resistance against the ICA, they did their tasks but nothing more than that.

### Process

#### Citizens in action

The main strategy of the ICA to engage citizens and patients was by means of concrete projects. A good example of this is the project ‘multi-problem families’, where families were engaged by the GP to participate. These families received care and/or support from multiple professionals. Each professional focussed on his/her own expertise and treatment goals, thereby failing to look at the family from a holistic point of view. This leads to chaos for the family with numerous professionals involved and the multiple, and sometimes conflicting, treatment goals. In the project, an independent coach from the ICA mapped out the main and overarching request for care with the families again. Often, care by some professionals could be stopped, preventing an uncontrollable stacking of care and making the care more transparent and efficient (‘engaging, innovation participants’).

We saw that if fellow citizens were enthusiastic about the ICA and Positive Health, this facilitated a positive attitude among other citizens. This was visible in the citizen science trajectory where the citizen scientists changed their name to ‘Health Ambassadors’ (‘engaging, opinion leaders’):

At first my neighbour was sceptical when I started talking about Positive Health and the ICA, but once we started talking and I told my [positive] experience, he also wanted to know more about Positive Health. (Interview citizen scientist, 2018)

#### Professionals in action

The projects in the ICA were developed ‘on the go’ and for each project a plan was developed according to a fixed format (‘planning’). However, room was given to the professionals and other stakeholders to refine the project if they thought it was more effective. The refinement of the original plan worked as a facilitator for implementation.

The professionals were initially engaged in the ICA by their own mother organisation. The engagement increased as more projects were developed over time. Some ICA projects even served as an example for other neighbourhoods outside the ICA. A sense of pride in the ICA was evoked by this facilitated engagement (‘engaging’):

The social team from [another neighbourhood] is going to work white label and they are using our ICA as a guideline. There are even social teams in other parts of Maastricht asking if they can work according to the principles of the ICA. This makes me proud. (Professional, interview 2019)

The actively engaged professionals were informal opinion leaders among the other professionals (construct ‘opinion leaders’). Additionally, the positive opinion of the participating GPs was seen as an informal influence on the opinion of other professionals (‘opinion leaders’).

Another strategy of the ICA was the formal appointment of two Positive Health coaches and one Positive Health ‘ambassador’ among the professionals working in the ICA (‘engaging champions’). These coaches and the ambassador were so enthusiastic about the ICA and Positive Health that they engaged other professionals in the sustainable use of Positive Health (Source: documents and observations, 2018–2020).

#### Combining budgets

Both the professionals and the other stakeholders were briefed on a regular basis about the progress of the ICA (‘reflecting and evaluating’). The daily board received a quarterly update on the progress of the ICA, the financial sponsors on a semi-annual basis. The professionals received a quarterly newsletter and received direct feedback from the project team members about the projects in which they were directly participating (Source: documents distributed from 2017–2020).

## Discussion

In this process evaluation, we described the facilitators and barriers of implementing an ICA in four deprived neighbourhoods using the CFIR. In this PHM initiative, several projects were developed ‘on the go’ during implementation between December 2016 and December 2020.

Overall, it took time to engage citizens in the ICA (core element ‘citizens in action’) as they initially perceived the ICA as externally developed, which was a barrier to implementation. Citizen engagement in the ICA through a citizen science trajectory facilitated implementation, not only because it revealed the needs of the citizens but it also stimulated an active contribution of citizens themselves. Our previous work showed that citizen science can empower citizens as active co-researchers instead of being passive research objects [22]. In the current study, we discovered that citizen scientists can also function as role models for their community. Role models can inspire individuals to adopt new behaviour and to stimulate group membership, which was important in the ICA since it helped to spread the ideology of Positive Health. However, the main strategy of the ICA to engage citizens and patients was through concrete projects. In the literature co-production between the patients and their professionals, co-designing of care is an important aspect in delivering quality care and improving population health [24].

On the level of professionals, we saw that the application and integration of Positive Health into the working routines worked as a facilitator for implementation. Positive Health functioned as a communication tool between the professionals and the citizens and between professionals themselves, as we saw in the construct ‘adaptability’. Wilderink et al. describe proper communication within and between organisations as a key element of a successful integrated community-based approach [25]. Although professionals were sceptical about the use of Positive Health at first, once they experienced the advantage of it, it became a facilitator. Nilsen et al. describe that organisational changes are more likely to succeed if professionals are being prepared for the change, there is prior communication about the implementation and if they have the opportunity to influence the change [26]. There was attention to all three elements in the ICA. Additionally, organisational change can bring perceived uncertainty about the work situation and role in an already high workload setting [27]. Having an influence on providing the bottlenecks in the delivery of care and welfare support was also, in the ICA, a facilitator for implementation as we saw in the construct ‘needs and resources of those served by the organisation’. Furthermore, the ‘tension for change’ worked as a facilitator for implementation since the professionals wanted to change the fragmented thinking among themselves and their colleagues in the health care landscape. To increase the feeling of ownership and to coach-on-the-job, the ICA appointed two enthusiastic professionals to help implement Positive Health. When the GPs started to openly communicate about the benefits of using Positive Health, the professionals became more engaged. The two coaches and the GPs served as informal role models for their colleagues. This was very important for the implementation of the ICA since it stimulated the use and joint language of Positive Health. In the construct ‘cosmopolitism’, we saw that collaboration between professionals of affiliated and non-affiliated organisations in the same neighbourhood, worked as a barrier since these professionals did not share the ideology of Positive Health.

For the core element ‘combining budgets’, the original idea of the ICA was to create one lump sum budget for both social and health care for all participating organisations, financed by the health insurer, Provincial State of Limburg and the municipality. Combining budgets was seen as an advantage by all stakeholders over the existing compartmented financial structure. It facilitated implementation of the ICA before the starting phase since it got everybody onboard. However, in the existing accounting and financial infrastructure, combing budgets was not achievable in the given timeframe (constructs ‘trialability’, ‘complexity’ and ‘implementation climate’) and became a barrier to implementation. Despite not reaching this goal, the stakeholders carried on developing the ICA regardless of not having one combined budget. In the construct ‘leadership engagement’, we saw that having a leader who went the extra mile to stimulate progress of the ICA worked as a facilitator. Additionally, only talking about pooled budgets can help to build trust to create a virtual combined budget in community programmes [28], which was also the case in this ICA.

This process evaluation contributes to existing literature on PHM initiatives since contextual factors and the mechanisms responsible for change in PHM initiatives are barely brought forward in most PHM definitions [2]. Furthermore, although PHM focusses on the wider range of determinants of health, the integration of professional and citizen perspectives, as in this paper, remains limited in the literature. We therefore advocate using a theoretical framework underlying the process evaluation to systematically assess the contextual factors in PHM initiatives and to facilitate comparison of the implementation process across PHM studies. We rated the constructs of the CFIR model on strength and valance of our study. To our knowledge, this has not been done before in a single case study.

Although structured guidelines and definitions are available on how to apply CFIR in implementation research [15], tailoring CFIR to the specific context and innovation at hand was challenging, considering the broadness of the framework. Varsi et al. state that the strength of the CFIR as a comprehensive framework may also be its weakness, since it may be too broad to capture all constructs with one data set [29]. We also experienced that it is difficult to analyse implicit factors with the CFIR. For example, ‘trust’ and ‘atmosphere’ are important aspects in the ICA but are not mentioned in the CFIR and, therefore, we coded them in other constructs such as ‘learning climate’. In the literature on collaborative governance, the implicit or ‘informal factors’ such as trust, commitment and shared understanding are also mentioned as important [30].

### Strengths and limitations

A strength of this study is that we followed the ICA for four years and collected myriad data from different perspectives, using various data collection techniques (data triangulation) and by applying a standardised framework. This increased the trustworthiness, credibility and validity of the study. A limitation is that, although the CFIR model in general fitted our research question and is comprehensive, there is still a risk that aspects were missed due to the broadness of the model. In addition, although rating the constructs can assist in getting an overview of the facilitators and barriers, it is also static, which is contradictory to a continuously changing implementation context.

## Conclusion

We conducted a process evaluation studying the facilitators and barriers of an ICA with the underlying theoretical framework of the CFIR. To our knowledge, this is the first time the CFIR was used to study the implementation of a PHM initiative. Engaging citizens and professionals at an early stage is an important facilitator for implementation. Additionally, the use of a shared vision on health worked as a facilitator since it created a shared language among professionals from different domains, which is an important aspect in PHM initiatives where multiple domains are expected to collaborate. Despite start-up problems in involving citizens and engaging professionals in the four neighbourhoods, and not creating a shared lump sum budget, the ICA continued to be implemented. Strong leadership, actively engaged citizens and professionals (opinion leaders), combined with a tension for change and insight into the needs of both citizens and professionals as a starting point for action were major facilitators for continued implementation, which helped in overcoming barriers.

## Supplemental Material

sj-docx-1-sjp-10.1177_14034948231199804 – Supplemental material for Implementation of an integrated community approach in deprived neighbourhoods: a theory-based process evaluation using the Consolidated Framework for Implementation Research (CFIR) Supplemental material, sj-docx-1-sjp-10.1177_14034948231199804 for Implementation of an integrated community approach in deprived neighbourhoods: a theory-based process evaluation using the Consolidated Framework for Implementation Research (CFIR) by SANNEKE J.M. GROOTJANS, M.M.N. STIJNEN, I. HESDAHL-DE JONG, M.E.A.L. KROESE, D. RUWAARD and M.W.J. JANSEN in Scandinavian Journal of Public Health
