# Supplementary material for: Implementation of an integrated community approach in deprived neighbourhoods: a theory-based process evaluation using the Consolidated Framework for Implementation Research (CFIR)
Source: Scand J Public Health. 2023 Sep 19;52(7):838–48. doi: 10.1177/14034948231199804 (PMC11481404; doi:10.1177/14034948231199804)
Supplement: sj-docx-1-sjp-10.1177_14034948231199804 – Supplemental material for Implementation of an integrated community approach in deprived neighbourhoods: a theory-based process evaluation using the Consolidated Framework for Implementation Research (CFIR) [file sj-docx-1-sjp-10.1177_14034948231199804.docx]

Contributions to literature in bulletpoint

- This study captures the facilitators and barriers of an implementation process of an integrated community approach (ICA) with eh use of the consolidated framework for implementation research (CFIR)
- Research has shown that there is a need for systematically assess the facilitators and barriers to enable successful implementation of complex interventions.
- We contribute to the existing literature on implementation science since the CFIR is not been used before to research the implementation of an ICA.
- This study uses a triangulation of data, which represents all users of the implementation, from citizens to financial sponsors.
